# Influence of type of uniform and days of usage in microbiological contamination of nurses uniform in a university hospital

**DOI:** 10.1186/1753-6561-5-S6-P317

**Published:** 2011-06-29

**Authors:** F Guillen-Grima, A Aguinaga-Perez, J Nunez-Cordoba, C Sara

**Affiliations:** 1 Health Sciences, Public University of Navarra, Spain; 2 Preventive Medicine, University of Vavarra Clinic, Pamplona, Spain; 3 Microbiology, University of Vavarra Clinic, Pamplona, Spain

## Introduction / objectives

Nurse uniforms can act as a reservoir of infections, with the areas around the pockets, cuffs and aprons the most contaminated. The aim of this study is compare the contamination of Standard nurse's uniform consisted of a dress, pinafore apron with the "scrub dress" type of uniform, as well as to measure the influence of the number of shifts as uniform was used in its contamination.

## Methods

Microbiological cultures were collected from uniforms of 88 nurses (58 using traditional uniform and 30 using scrub dress) in an university hospital, during their work in one month period. Cultures were obtained using Count-tact plates (BioMérieux) plates (25 cm2) The culture media were incubated for five days at room temperature. After the incubation period bacterial count and type of bacteria colonizing the uniform were evaluated by microbiologist. Reading was provide in cfu colony forming units. Student T , and correlation were computed with SPSS v.17.

## Results

The average count was 42,82 fcu/cm2 . There was no differences in the count between both types of uniform.(P=0,504). There was a positive correlation between the number of days and microbiological count. (r=0,224, P=0,036). The average count was 44,36 cfu/cm in those nurses using the uniform for 1 or 2 shifts, and 65,20cfu this difference was statistically significant. (P=0,031).

## Conclusion

There are no differences in microbiological contamination between standard or scrub uniform. The main differences in contamination appeared in those nurses that used the same uniform for more than 2 shifts. Hospital should provided nurses with enough uniforms to change before every shift or at least every 2 shifts.

## Disclosure of interest

None declared.

